# Pro-Oxidant Activity of an ALS-Linked SOD1 Mutant in Zn-Deficient Form

**DOI:** 10.3390/molecules25163600

**Published:** 2020-08-07

**Authors:** Chise Nagao, Kunisato Kuroi, Taiyu Wakabayashi, Takakazu Nakabayashi

**Affiliations:** 1 Faculty of Pharmaceutical Sciences, Tohoku University, Sendai 980-8578, Japan; chise.nagao.t3@dc.tohoku.ac.jp (C.N.); kkuroi@pharm.kobegakuin.ac.jp (K.K.); 2 Graduate School of Pharmaceutical Sciences, Tohoku University, Sendai 980-8578, Japan; wakabayashit333@gmail.com; 3 Faculty of Pharmaceutical Sciences, Kobe Gakuin University, 1-1-3 Minatojima, Chuo-ku, Kobe 650-8586, Japan

**Keywords:** SOD1, ALS, cytotoxicity, pro-oxidant activity, copper, zinc

## Abstract

Cu, Zn superoxide dismutase (SOD1) is a representative antioxidant enzyme that catalyzes dismutation of reactive oxygen species in cells. However, (E,E)-SOD1 mutants in which both copper and zinc ions were deleted exhibit pro-oxidant activity, contrary to their antioxidant nature, at physiological temperatures, following denaturation and subsequent recombination of Cu^2+^. This oxidative property is likely related to the pathogenesis of amyotrophic lateral sclerosis (ALS); however, the mechanism by which Cu^2+^ re-binds to the denatured (E,E)-SOD1 has not been elucidated, since the concentration of free copper ions in cells is almost zero. In this study, we prepared the (Cu,E) form in which only a zinc ion was deleted using ALS-linked mutant H43R (His43→Arg) and found that (Cu,E)-H43R showed an increase in the pro-oxidant activity even at physiological temperature. The increase in the pro-oxidant activity of (Cu,E)-H43R was also observed in solution mimicking intracellular environment and at high temperature. These results suggest that the zinc-deficient (Cu,E) form can contribute to oxidative stress in cells, and that the formation of (E,E)-SOD1 together with the subsequent Cu^2+^ rebinding is not necessary for the acquisition of the pro-oxidant activity.

## 1. Introduction

Amyotrophic lateral sclerosis (ALS) is a progressive neurodegenerative disease in which the loss of motor neurons causes a decrease in, for example, muscular strength and respiratory function, eventually leading to death. Neither the onset mechanism nor a fundamental treatment for ALS have been established [1,2,3]. More than 20 causative genes and proteins, including TAR DNA-binding protein 43 (TDP-43), fused in sarcoma (FUS), C9orf72, and ataxin-2, have been identified by recent genetic analysis of ALS patients and pathological examination of patient’s neurons, and these genes and proteins have been extensively investigated in terms of the pathology of ALS [4,5,6,7,8,9,10,11]. Mutants of Cu, Zn superoxide dismutase (SOD1) were first identified as causative proteins [12,13].

**Figure 1:**
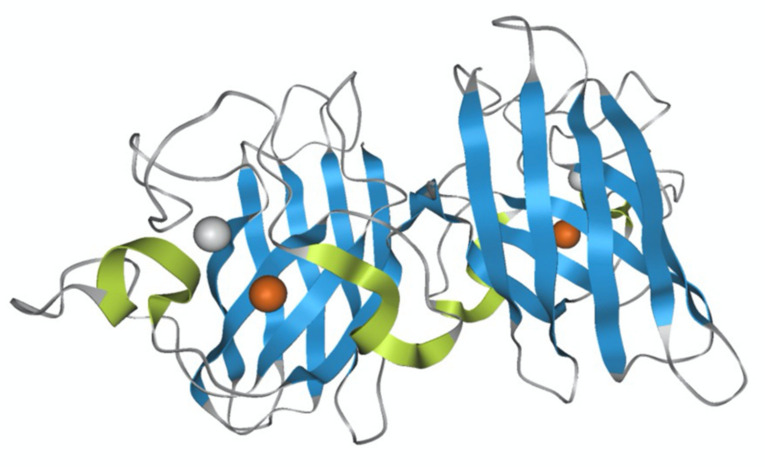
Molecular structure of wild-type superoxide dismutase (SOD1). PDB 1PU0. The blue is the eight anti-parallel ß strands, and the yellow-green is the three loops that connect them. The metal is Zn in white and Cu in brown.

Native SOD1 is a metal-binding protein containing one Cu ion and one Zn ion in the metal-binding region, and its monomer has a molecular weight of 16 kDa (Figure 1). SOD1 typically forms a stable homodimer that functions as an antioxidant enzyme and converts superoxide anion, a reactive oxygen species (ROS), into hydrogen peroxide. The coordinated Cu ion is a catalyst for this anti-oxidant activity, whereas the coordinated Zn ion acts to stabilize its molecular structure. More than 180 SOD1 gene mutations have been identified in familial ALS patients, and SOD1-positive aggregates have been found, suggesting a relationship between SOD1 and the onset of ALS [14]. A proposed mechanism for ALS onset by SOD1 mutants suggests that SOD1 becomes cytotoxic, inducing the pathogenesis of ALS. Several hypotheses for SOD1 cytotoxicity have been reported, including the aggregate hypothesis in which SOD1 forms aggregates in nerve cells to induce cell death [15,16,17,18,19], and the oxidative stress hypothesis in which SOD1 exhibits pro-oxidant activity [12,20,21,22,23]. A hypothesis that SOD1 mutants increase endoplasmic reticulum stress has also been proposed to be involved in the onset of ALS [24]. Furthermore, misfolded wild-type (WT) SOD1 has been detected in ALS patients and thus may contribute to cytotoxicity, inducing ALS [25,26].

We have systematically investigated the acquisition of the pro-oxidant activity of denatured SOD1, a behavior that is opposite to its usual dismutation function [27,28,29,30,31,32]. We showed that the metal-depleted (apo) SOD1 mutants ((E,E)-SOD1, hereafter SOD1 is referred to as (m_1_,m_2_)-SOD1, where (m_1_,m_2_) means coordinated metal ions in the Cu- and Zn-binding sites, respectively, and E is empty) were unfolded at 37 °C and these denatured SOD1 exhibited the pro-oxidant activity after rebinding Cu^2+^. Hydroxyl radical, very harmful ROS species, was generated from the reaction of H_2_O_2_ with the denatured SOD1 mutants [32]. The pro-oxidant activity was greatest when Cu ions were re-bound to both the Cu and Zn-binding sites in the denatured SOD1. The Cu ion in the Cu-binding site is responsible for the pro-oxidant activity, and that in the Zn-binding site has an auxiliary function to enhance the pro-oxidant activity [32]. It is worth noting here that denaturation temperature of metallated (holo) SOD1 ((Cu,Zn)-SOD1) is above 75 °C [33], and denaturation of (Cu,Zn)-SOD1 does not occur at physiological temperatures.

Some ALS-linked SOD1 mutants can be denatured at a physiological temperature of 37 °C when the metal ions are removed to be their (E,E) forms [27,28,29,30,31,32]. The aggregation of ALS-linked SOD1 mutants at physiological temperatures also occurs only after the demetallation and the subsequent denaturation [19]. Demetallation to form (E,E)-SOD1 is therefore required for the ALS-linked SOD1 mutants to be denatured at physiological temperatures, resulting in the pro-oxidant activity and the aggregation.

The pro-oxidant activity was observed for (E,E)-SOD1, irrespective of mutation, following the denaturation and the subsequent re-binding of Cu^2+^ [27,28,29,30,31,32]. The finding of metal-depleted SOD1 in ALS model mice [34] supports a model in which (E,E)-SOD1 is a precursor to cytotoxic expression. However, since the intracellular Cu concentration is strictly controlled, and the concentration of free Cu ion is extremely low in living cells [35], the mechanism by which Cu^2+^ rebinds to the denatured (E,E)-SOD1 in cells remains unclear. On the other hand, it has been reported that Zn transporter expression was reduced and the homeostasis of Zn^2+^ was disrupted in ALS patients [36,37]. This could result in Zn deficiency in SOD1 in cells of ALS patients, leading to the formation of Zn-deficient SOD1 in which only a Cu ion is bound ((Cu,E)-SOD1). As mentioned above, the coordinated Zn ion contributes to stabilization of the protein structure [38,39], and thus the structural stability of (Cu,E)-SOD1 is likely reduced due to the deficiency of a Zn ion. Thus, if (Cu,E)-SOD1 shows the pro-oxidant activity at physiological temperatures, this would suggest a mechanism in which the pro-oxidant activity is obtained without the (E,E)-SOD1 formation and the Cu^2+^ rebinding in cells. In the present study, we therefore prepared (Cu, E) species using H43R (His43→Arg), which is classified into a group of ALS-linked mutants causing fast progression of ALS, and evaluated its structural stability and pro-oxidant activity.

## 2. Results and Discussion

**Figure 2:**
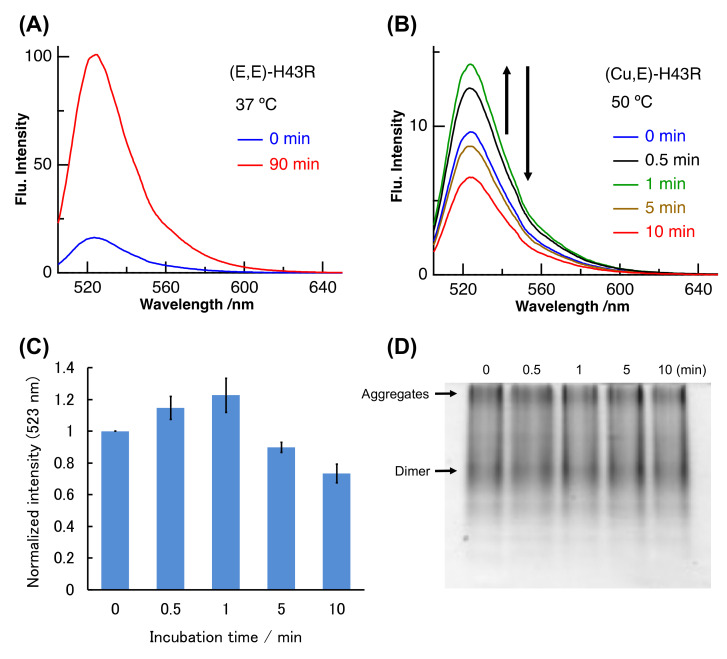
(**A**–**C**) Pro-oxidant activities after different incubation times in phosphate buffer. (**A**): (E,E)-H43R at 37 °C, (**B**): (Cu,E)-H43R at 50 °C. The dichlorofluorescein (DCF) fluorescence intensities normalized by the value at 0 min for (**B**) are shown in (**C**). Error bars are SE (*n* = 3). Pro-oxidant activity was measured by the fluorescence intensity of DCF generated by the oxidation of dichlorodihydrofluorescein (DCFH) (50 µM) by protein (10 µM) and H_2_O_2_ (50 µM) at room temperature. CuCl_2_ (20 µM) was also added in (A) before the fluorescence measurement. (**D**) Native-poly acrylamide gel electrophoresis (PAGE) of (Cu,E)-H43R in phosphate buffer after the indicated incubation times.

(Cu,E)-H43R was prepared by dialyzing (Cu, Zn)-H43R. H43R as the (Cu, E) form was evaluated to comprise 40~70% of the total H43R in the prepared solution (see Materials and Methods). The binding constant of Cu ion to SOD1 is very large, and the log of the binding constant of H43R is in 12.9–18.8 even in the denature condition [29,32]. We first investigated the pro-oxidant activity of the prepared (Cu,E)-H43R after the denaturation using the dichlorofluorescein (DCF) fluorescence method. In this assay, non-fluorescent 2′,7′-dichlorodihydrofluorescein (DCFH) is added to solution of H_2_O_2_ and SOD1. Fluorescent DCF is then generated by the oxidation reaction between DCFH and ROS generated from H_2_O_2_ catalyzed by SOD1. The DCF fluorescence intensity therefore reflects the pro-oxidant activity of SOD1 [27,28,29,30,31,32]. Figure 2A shows the example of the increase in the DCF fluorescence due to the pro-oxidant activity. (E,E)-H43R acquires the pro-oxidant activity after the incubation at 37 °C and subsequent addition of Cu^2+^. As shown in Figure 2A, the DCF fluorescence of (E,E)-H43R strongly increased after 90 min incubation at 37 °C and subsequent Cu^2+^ addition, indicating that the pro-oxidant activity was acquired following the denaturation and Cu^2+^ rebinding [27,28,29,30,31,32].

We measured the DCF fluorescence due to (Cu,E)-H43R at 50 °C (Figure 2B). The DCF fluorescence intensity increased with incubation time (0–1 min), then decreased gradually (1–10 min). This result indicates that (Cu,E)-H43R also acquired the pro-oxidant activity following the incubation at 50 °C, but this activity was much lower than that of the denatured (E,E)-H43R that subsequently bound Cu^2+^ at 37 °C (Figure 2A). Figure 2C shows the fluorescence intensities due to (Cu,E)-H43R, normalized by the fluorescence intensity at 0 min. The pro-oxidant activity was convex-shaped, first increasing and then decreasing with incubation time.

We used native-poly acrylamide gel electrophoresis (PAGE) to determine whether the decrease in the pro-oxidant activity of (Cu,E)-H43R is due to aggregation (Figure 2D). Native-PAGE is performed without adding a reducing agent or surfactant, allowing estimation of the quaternary structure of a protein because the protein migrates while maintaining its three-dimensional structure. Note that the electrophoresis distance does not strictly reflect molecular weight. Bands due to both the dimer species and aggregates were observed near the center and at the top of the gel, respectively, and their concentrations seemed to remain unchanged irrespective of the incubation time. This result indicates that the decrease in the pro-oxidant activity of (Cu,E)-H43R incubated at 50 °C was not due to aggregation at the present incubation times.

**Figure 3:**
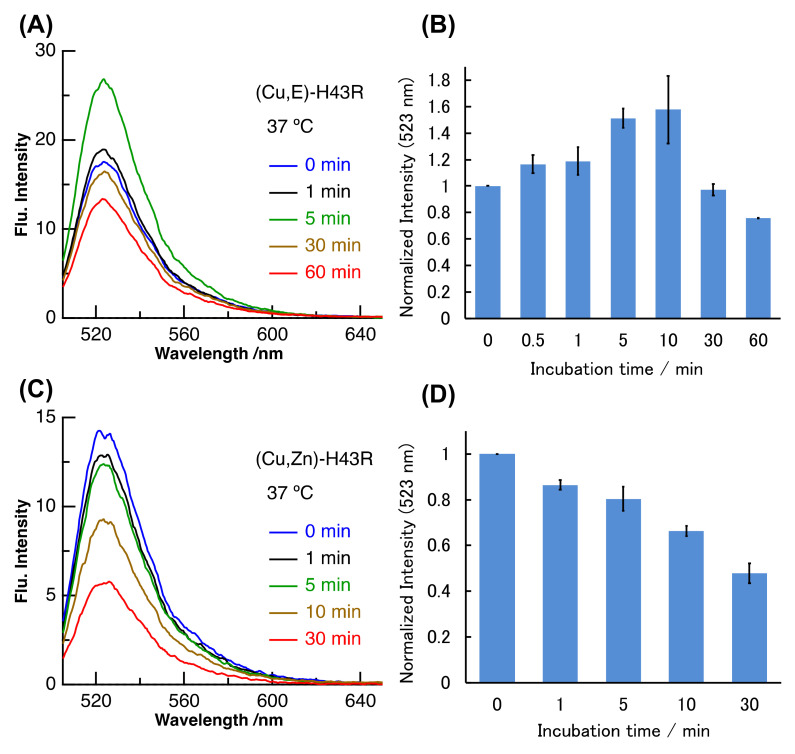
Pro-oxidant activities after different incubation times in phosphate buffer. (**A**,**B**): (Cu,E)-H43R at 37 °C, (**C**,**D**): (Cu,Zn)-H43R at 37 °C. The DCF fluorescence intensities normalized by the value at 0 min for (**A**) and (**C**) are shown in (**B**) and (**D**), respectively. Error bars are SE (*n* = 3). Pro-oxidant activity was measured by the fluorescence intensity of DCF generated by the oxidation of DCFH (50 µM) by protein (10 µM) and H_2_O_2_ (50 µM) at room temperature.

**Figure 4:**
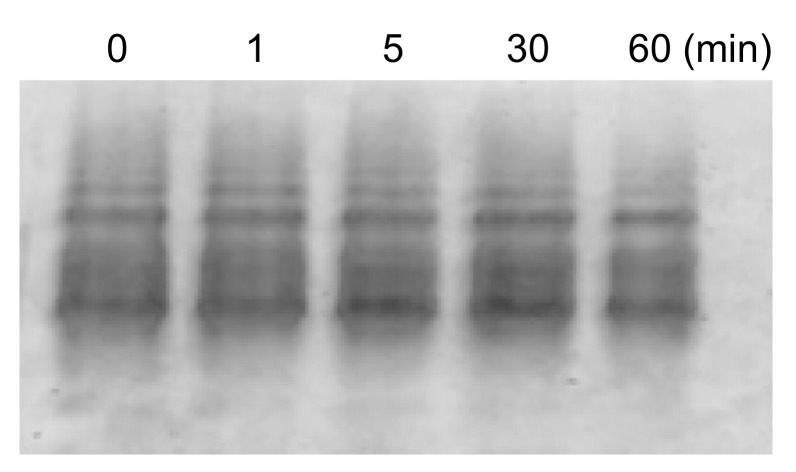
Native-PAGE of (Cu,E)-H43R in phosphate buffer after the indicated incubation times at 37 °C.

High temperatures such as 50 °C likely induce structural changes in (Cu,E)-H43R, resulting in the increase in the pro-oxidant activity. However, since high temperature of 50 °C does not occur in vivo, the same experiment was performed at a physiological temperature (37 °C; Figure 3A,B). Incubation at 37 °C in buffer solution also increased the DCF fluorescence intensity of (Cu,E)-H43R, leading to the conclusion that (Cu,E)-H43R also acquired the pro-oxidant activity after the incubation at physiological temperature. The pro-oxidant activity at 37 °C increased slowly compared to that at 50 °C, reaching a maximum at 5–10 min and then decreased with incubation time. As shown in Figure 3C,D, only a decrease in the DCF fluorescence intensity with incubation time was observed for (Cu,Zn)-H43R, confirming that the increase in the pro-oxidant activity of (Cu,E)-H43R arises from the deficiency of a Zn ion. As is the case of 50 °C, the decrease in the pro-oxidant activity with incubation time was observed both for (Cu,E)- and (Cu,Zn)-H43R at 37 °C, and no aggregation of (Cu,E)-H43R with increasing incubation time was observed in native-PAGE (Figure 4).

**Figure 5:**
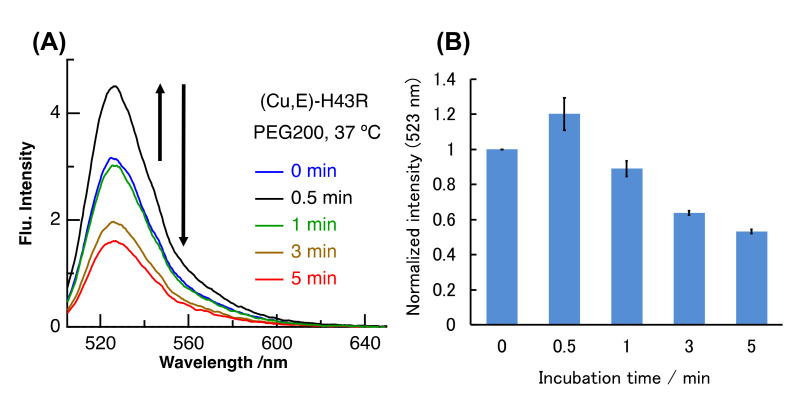
(**A**) Pro-oxidant activities of (Cu,E)-H43R at 37 °C after different incubation times in 20 wt% PEG200 solution. (**B**) DCF intensities normalized by the value at 0 min for (**A**). Error bars are SE (*n* = 3). Pro-oxidant activity was measured by the fluorescence intensity of DCF generated by the oxidation of DCFH (50 µM) by protein (10 µM) and H_2_O_2_ (50 µM) at room temperature.

We have further investigated the pro-oxidant activity of (Cu,E)-H43R in macromolecular crowding environment mimicking in vivo conditions. The intracellular environment is highly crowded with biomolecules such as proteins and lipids, and this condition is called macromolecular crowding. The structures and functions of some proteins and nucleic acids in cells are different compared to those in buffer solution due to macromolecular crowding [40,41,42]. WT SOD1 is very stable compared with ALS-linked mutants and is not denatured even in its (E,E) form at 37 °C in buffer solution. However, we very recently reported that (E,E)-WT also showed the denaturation and the subsequent pro-oxidant activity at 37 °C in molecular crowding conditions that mimic intracellular environment by the addition of polyethylene glycol (PEG) into buffer solution [31]. Molecular crowding environment is thus one factor that induces the structural instability of SOD1. We therefore investigated the properties of (Cu,E)-H43R when incubated at 37 °C under the molecular crowding environment mimicked by PEG. As shown in Figure 5, the DCF fluorescence intensity due to (Cu,E)-H43R at 37 °C in the molecular crowding environment increased and then gradually decreased with incubation time. This result indicates that (Cu,E)-H43R also exhibits the increase in the pro-oxidant activity under the molecular crowding environment at 37 °C. The incubation time when the pro-oxidant activity reached the maximum was ~0.5 min in Figure 5, which is much faster than that at 37 °C in buffer solution (Figure 3), and is similar to that at 50 °C (Figure 2).

The present study demonstrates that (Cu,E)-H43R acquires the pro-oxidant activity with incubation at physiological temperature. However, the gradual decrease in the pro-oxidant activity was also observed in all the present experimental conditions. One possible explanation for the decrease in the pro-oxidant activity is the formation of aggregates, but native-PAGE showed that the amount of protein aggregates did not increase with incubation time. We thus hypothesized that the observed reduction in the pro-oxidant activity with incubation time resulted from the desorption of a Cu ion from SOD1. Since the binding of a Cu ion is necessary for SOD1 to exert the pro-oxidant activity, the removal of a Cu ion from (Cu, E)-H43R results in the loss of its pro-oxidant activity.

**Figure 6:**
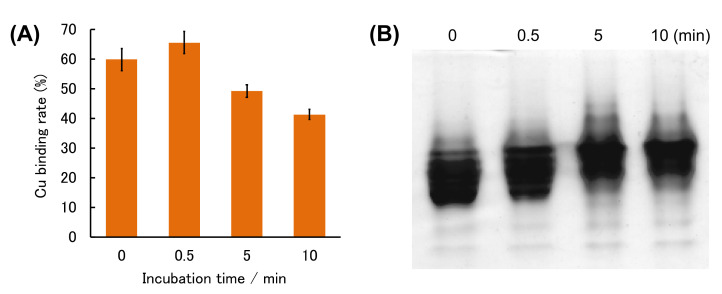
(**A**) Cu binding rate of (E,E)-H43R with one equivalent of Cu^2+^ during incubation at 50 °C in phosphate buffer. Error bars are SE (*n* = 3). (**B**) Native-PAGE of (E,E)-H43R with one equivalent of Cu^2+^ in phosphate buffer after the indicated incubation times.

We thus prepared (Cu,E)-H43R by adding one equivalent of Cu^2+^ to solution of (E,E)-H43R, and measured the change in the amount of Cu ions bound to H43R with incubation time. The 4-(2-pyridylazo)-resorcinol (PAR) method was used to estimate the amount of the coordinated Cu ions. PAR is a chelator that exhibits visible absorption upon binding Cu^2+^ (PAR-Cu^2+^ complex). Sequential addition of two strong chelators, nitrilotriacetic acid (NTA) and ethylenediaminetetraacetic acid (EDTA), to the PAR solution removes Cu^2+^ from PAR and reduces visible absorption due to the PAR-Cu^2+^ complex. Therefore, measurement of the difference in absorbance before and after the addition of NTA and EDTA allows for the estimation of the concentration of Cu^2+^ in the sample solution based on a calibration curve prepared with Cu^2+^ solutions of known concentrations [29,43]. The amount of Cu ions bound to SOD1 in the (Cu,E)-H43R solution decreased by ~20% after 10 min incubation at 50 °C (Figure 6A), corresponding to the decrease in the pro-oxidant activity shown in Figure 2. This result implies that the gradual decrease in the pro-oxidant activity is partly due to the desorption of Cu ions from H43R. Note that (Cu,E)-H43R used in Figure 6 was prepared from (E,E)-H43R, which is different from (Cu,E)-H43R prepared without prior formation of its (E,E) form in Figure 2, Figure 3, Figure 4 and Figure 5. The effect of the apo formation is apparently not negligible. The reduced binding rate of Cu ions to (Cu,E)-H43R in Figure 6A is not due to aggregate formation because no aggregation was detected by native-PAGE for (Cu,E)-H43R prepared via the (E,E) form with the incubation (Figure 6B).

**Figure 7:**
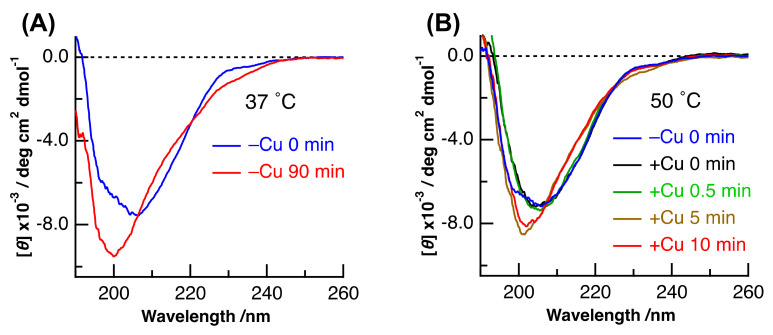
(**A**) CD spectra of (E,E)-H43R before and after 90 min incubation at 37 °C in phosphate buffer. (**B**) CD spectra of (E,E)-H43R and of (Cu,E)-H43R prepared by the addition of one equivalent of Cu^2+^ to (E,E)-H43R at different incubation times. The incubation temperature of (**B**) was 50 °C.

CD spectra of (Cu,E)-H43R with different incubation times were measured to examine the time-dependent change in the secondary structure. We previously showed that the denaturation of SOD1 can be confirmed by the change in its CD spectrum [27,28,29,30,31,32]. As is the case of (E,E)-H43R at 37 °C in Figure 7A, the CD spectrum exhibits a decrease and an increase in intensity at around 215 and 225 nm, respectively, with incubation time, which results from the decrease in β-sheet and turn structures and the increase in irregular structures in (E,E)-H43R. The irregular structure was estimated to increase by 15–20% [27] after the 90 min incubation at 37 °C.

Figure 7B shows the CD spectra of (Cu,E)-H43R with different incubation times, together with that of (E,E)-H43R before the addition of Cu^2+^. The incubation temperature was 50 °C. The shape of the CD spectrum of (E,E)-H43R was different before and just after the addition of Cu^2+^. Furthermore, a subsequent change in the CD spectrum after 5 min incubation led to a third shape. The pro-oxidant activity of (Cu,E)-H43R at 50 °C increased during 0-1 min incubation, then decreased after 5 min incubation (Figure 2). Thus, the relationship between the results of the CD and the pro-oxidative activity could be explained as follows: the structure of (E,E)-H43R is slightly changed by Cu coordination, and this state exhibits the increase in the pro-oxidant activity; then, subsequent structural changes result in the decrease in the pro-oxidant activity, in part due to Cu^2+^ desorption from the protein. The CD spectrum of (Cu,E)-H43R after 0–0.5 min incubation was different from that of the denatured (E,E)-H43R after 90 min incubation (Figure 7A), and was similar to that of (Cu,Zn)-H43R [27]. This means that the secondary structure of (Cu,E)-H43R is almost the same as that of (Cu,Zn)-H43R, and slight decrease in irregular structure occurs with the addition of Cu^2+^ to (E,E)-H43R because the content of irregular structure was estimated to be larger in (E,E)-H43R at 0 min incubation than in (Cu,Zn)-H43R [27]. This result suggests that a partial change in the coordination structure around a Cu ion induces the pro-oxidant activity of (Cu, E)-H43R.

**Figure 8:**
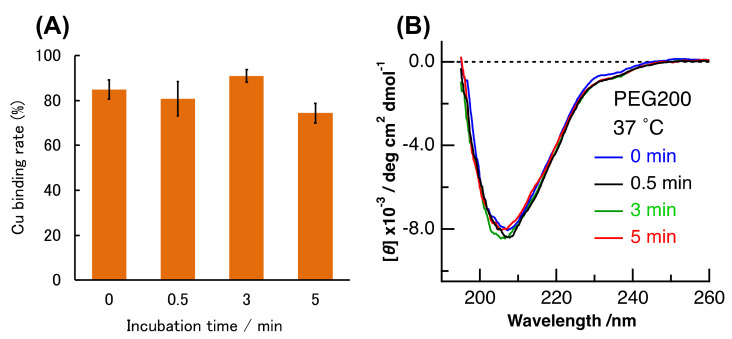
(**A**) Cu binding rate of (E,E)-H43R with one equivalent of Cu^2+^ during incubation at 37 °C in phosphate buffer containing 20 wt% PEG200. Error bars are SE (*n* = 3). (**B**) CD spectra of (E,E)-H43R with one equivalent of Cu^2+^ after different incubation times in 20 wt% PEG200 solution. The incubation temperature in (**B**) was 37 °C.

Similar experiments were conducted in crowding environment. PAR and CD measurements were performed for (Cu, E)-H43R prepared from (E,E)-H43R, as mentioned above in the presence of PEG200 at 37 °C, and the results are shown in Figure 8. The shape of the CD spectrum remained unchanged with time within experimental uncertainty (Figure 8B), and the amount of bound Cu ions slightly decreased by ~10% after 5 min incubation (Figure 8A). The shape of the CD spectrum in crowding environment is the same as that in buffer solution after 0–0.5 min incubation at 50 °C (Figure 7B), which is assigned to the partial structural change. As mentioned above, the pro-oxidant activity in crowding environment is convex-shaped, first increasing and then decreasing with incubation time (Figure 5B), which is the same as the case in buffer solution at 50 °C (Figure 2C). Therefore, the partial structural change induced the increase in the pro-oxidant activity of (Cu,E)-H43R in both the conditions: at 50 °C, and in a crowding environment. It seems also to be consistent with the decrease in the pro-oxidant activity in response to the loss of coordinated Cu ions (Figure 8A), although the magnitude of the Cu-desorption is smaller in molecular crowding environment.

The denatured (E,E)-SOD1 exhibits strong pro-oxidant activity after Cu^2+^ rebinding and retains this activity to some extent. However, (Cu,E)-H43R has different properties from its (E,E) form, initially exhibiting low pro-oxidant activity that gradually decreases, partly due to the Cu-desorption. The CD spectra of the (E,E) and (Cu,E) forms are different when their pro-oxidant activities are high, implying that the difference in the pro-oxidant activity is due to the difference in the protein structure.

## 3. Materials and Methods

### 3.1. Sample Preparation

Expression of human SOD1 in *Escherichia coli* and its purification were carried out in the same procedure as described previously [27,28,29,30,31,32]. Cleavage of His-tag from protein was performed by treatment with thrombin (GE Healthcare, Chicago, IL, USA) at 22 °C for 16 h and then an immobilized metal affinity chromatography column charged with Ni^2+^ (GE Healthcare, Chicago, IL, USA). Isolated His-tag-removed protein was used in all the experiments.

(E,E)-H43R was obtained by dialysis using EDTA from (Cu,Zn)-H43R [27,28,29,30,31,32]. In the preparation of the (Cu, E) form, first, a small amount of acetic acid was added to the (E,E)-H43R solution to adjust pH 3.8, and 1 equivalent of Zn and 4 equivalents of Cu were added and mixed to the protein solution. Next, the protein solution was dialyzed at 4 °C for 7 h in an acetate buffer (pH 3.8) as a dialysis solution, and then was dialyzed in a phosphate buffer (pH 7.4) at 4 °C for 1.5 h. The ratio of the obtained (Cu, E) form to the protein concentration was estimated to 40~70% by the PAR method, which was used as the (Cu, E)-H43R solution in the subsequent experiments.

The PAR method for estimating the metal-binding ratio in H43R was performed as follows. PAR solution was first prepared by adding 100 µM PAR to a borate/hydrochloric acid buffer containing 6 M guanidine-HCl that had been chelated to completely remove trace Cu and Zn ions in advance. Twenty microliters of the sample solution was added into the PAR solution, and then time-lapse measurements of absorbance at 500 nm were initiated. After the time change in absorbance was no longer observed, 0.8 mM NTA was added and absorbance measurements were continued. Then, after almost no time change in absorbance was again observed, 0.8 mM EDTA was added and absorbance was measured over time. This measurement was performed for several CuCl_2_ or Zn(CH_3_COO^–^)_2_ aqueous solutions with known concentrations to prepare a calibration curve, and then the same operation was performed for the aqueous solution containing H43R to determine the metal concentration in the H43R solution. In this assay, H43R was completely denatured in advance by 6 M guanidine-HCl to remove metal ions bound to H43R, and NTA and EDTA were regarded to chelate Zn^2+^ and Cu^2+^, respectively, in the measurement of the H43R solution. The number of Cu^2+^ and Zn^2+^ ions per H43R monomer was estimated from the ratio of a metal ion to the protein concentration. The analysis of Cu binding rate of (Cu,E)-H43R during incubation was performed three times with independent samples, and the average with its standard error was used at each incubation time. The concentration of H43R was evaluated using a bicinchoninic acid assay or absorbance at 280 nm and was given in monomer unit throughout in this paper.

### 3.2. Analyses of Sample Solution

The pro-oxidant activity of H43R was measured by a fluorescence assay using 2,7-dichlorofluorescein (DCF) [27,28,29,30,31,32]. 2,7-dichlorodihydrofluorescein (DCFH), H_2_O_2_, and (Cu, E)-H43R were mixed in 50 mM phosphate buffer (pH 7.4), and the final concentrations were 50 µM, 50 µM, and 10 µM, respectively. After the mixed solution was shielded from light for 5 min at room temperature, the fluorescence spectrum was measured with the excitation wavelength of 495 nm. The peak fluorescence intensity at 523 nm was used as the magnitude of the pro-oxidant activity. The experiment was performed three times with independent samples, and the average with its standard error was used at each incubation time.

Native-PAGE was performed under the condition without the addition of both a reducing agent and a surfactant. The final concentration of the protein was 16~20 µM. CD spectra were recorded by a spectropolarimeter J-820 (Jasco, Tokyo, Japan) and a quartz cell with a 0.5 mm path length. The final concentration of the protein was 20 µM, and the spectrum obtained by the average of four spectra was shown as a representative spectrum. Absorption spectra were measured by a U-3300 spectrophotometer (Hitachi, Tokyo, Japan) using a quartz cell with 10 mm path length, and the fluorescence spectra were measured on a FP-6300DS (Jasco, Tokyo, Japan) using a 3 × 3 mm^2^ quartz cell.

## 4. Conclusions

Previous studies have shown that the denaturation of (E,E)-SOD1, followed by the rebinding of Cu^2+^ results in the acquisition of strong pro-oxidant activity [27,28,29,30,31,32]. Metal-depleted SOD1 was observed in cells [34]; however, due to the almost zero intracellular concentration of free copper ion, the mechanism for the recombination of copper ions to the denatured (E,E)-SOD1 was unclear. The present study shows that (Cu,E)-H43R exhibits the increase in the pro-oxidant activity, indicating that the (E,E)-SOD1 formation and the Cu^2+^ rebinding are not necessary for the increase in the pro-oxidant activity. The pro-oxidant activity of (Cu,E)-H43R also increases even at 37 °C in PEG200 solution that mimics intracellular environment, implying that (Cu,E)-H43R can exert toxicity in vivo. Here, we propose a mechanism for SOD1-induced oxidative stress in which the Zn-deficient (Cu, E) forms contribute to oxidative stress.
